# Supplementary material for: Salvage Radical Prostatectomy for Recurrent Prostate Cancer: A Systematic Review (French ccAFU)
Source: Cancers (Basel). 2023 Nov 20;15(22):5485. doi: 10.3390/cancers15225485 (PMC10670522; doi:10.3390/cancers15225485)
Supplement: Supplementary file 1 [file cancers-15-05485-s001.zip › cancers-2693525-supplementary.pdf]

Study objective	Number of included retrospective studies fulfilling the criterion
1. Is the hypothesis/aim/objective of the study stated clearly in the abstract, introduction, or methods section?	55/55
**Study population**	
2. Are the characteristics of the participants included in the study described?	55/55
3. Were the cases collected in more than one centre?	9/55
4. Are the eligibility criteria (inclusion and exclusion criteria) for entry into the study explicit and appropriate?	51/55
5. Were participants recruited consecutively?	53/55
6. Did participants enter the study at a similar point in the disease?	0/55
**Intervention and co-intervention**	
7. Was the intervention clearly described in the study?	55/55
8. Were additional interventions (co-interventions) clearly reported in the study?	0/55
**Outcome measure**	
9. Are the outcome measures clearly defined in the introduction or methods section?	55/55
10. Were relevant outcomes appropriately measured with objective and/or subjective methods?	55/55

11. Were outcomes measured before and after intervention?	55/55
**Statistical analysis**	
12. Were the statistical tests used to assess the relevant outcomes appropriate?	48/55
**Results and conclusions**	
13. Was the length of follow-up reported?	51/55
14. Was the loss to follow-up reported?	0/55
15. Does the study provide estimates of the random variability in the data analysis of relevant outcomes?	0/55
16. Are adverse events reported?	55/55
17. Are the conclusions of the study supported by results?	55/55
**Competing interests and sources of support**	
18. Are both competing interests and sources of support for the study reported?	52/55

Table S1: Risk of bias in non-randomized trials using the ROBINS-I tool
